# Ageism and Behavior Change During a Health Pandemic: A Preregistered Study

**DOI:** 10.3389/fpsyg.2020.587911

**Published:** 2020-11-19

**Authors:** Michael T. Vale, Jennifer Tehan Stanley, Michelle L. Houston, Anthony A. Villalba, Jennifer R. Turner

**Affiliations:** Department of Psychology, University of Akron, Akron, OH, United States

**Keywords:** COVID-19, ageism, hostile ageism, benevolent ageism, behavior change, attitudes

## Abstract

The COVID-19 pandemic has led to a suspected surge of ageism in America and has imposed critical health and safety behavior modifications for people of all ages (Ayalon et al., 2020; Lichtenstein, 2020). Given that older adults are a high-risk group, maintaining their safety has been paramount in implementing preventive measures (i.e., more handwashing, social distancing); however, making such behavior modifications might be contingent on how one views older adults (i.e., ageist stereotypes). Therefore, the goal of the current pre-registered study was to explore if hostile and benevolent ageism relate to pandemic-related fear and behavior change. An online survey assessing responses to the pandemic was taken by 164 younger and 171 older adults. Higher hostile ageism predicted lower pandemic-related behavior modification. Those high in benevolent ageism reported lower behavior change, but also reported higher pandemic-related fear; however, when pandemic-related fear was considered a mediator between the two, the directionality between benevolent ageism and behavior change switched, indicating a suppression effect. These findings highlight that ageist attitudes do predict responses to the pandemic and that hostile and benevolent ageism are distinct facets that have unique implications during a health pandemic.

## Introduction

The COVID-19 pandemic has altered nearly all facets of everyday life for Americans, imposing critical hygiene and safety related behavior modifications that are associated with high levels of stress (American Psychological Association, 2020). More specifically, preventive methods recommended by the Center for Disease Control and Prevention (CDC) consist of physical distancing, increased handwashing, regular disinfection of commonly touched surfaces, and cessation of non-essential travel, especially for vulnerable populations such as older adults (Center for Disease Control, 2020). Maintaining the safety of older adults during the pandemic has been emphasized in an effort to motivate the publics’ willingness to engage in safety precautions, despite warnings that such dealings can agitate intergenerational tensions and motivate increased ageism (Ayalon, 2020; Ayalon et al., 2020; Brooke and Jackson, 2020; Cesari and Proietti, 2020; Fraser et al., 2020; Lichtenstein, 2020; Morrow-Howell et al., 2020; Petretto and Pili, 2020; Rahman and Jahan, 2020). Overt and covert forms of ageism are purported to be embedded in the public’s response and perception of the pandemic and range from: the antagonistic *#BoomerRemover* tag, to undermining of older adults’ independence in making health-related decisions, and the incorrect portrayal of older adults as a homogenous group (Ayalon, 2020; Ayalon et al., 2020; Fraser et al., 2020; Lichtenstein, 2020). However, ageist ideology has yet to be empirically associated with responses and attitudes toward the pandemic. The purpose of this paper is to explore if ageist attitudes, both benevolent and hostile, relate to individuals’ behavioral responses to the pandemic.

Ageism refers to the prejudice directed at people because of their perceived age and covers the multilayered configuration of stereotypes and discrimination directed toward older adults (Nelson, 2016; Cary et al., 2017). Classic definitions of ageism stipulate that older people are viewed in an undesirable fashion; however, attitudes toward older adults are more complex and are similar to sexist depictions of women, such that they both fit the paternalistic stereotype and are simultaneously viewed as being warm, but incompetent, resulting in both hostile and benevolent forms of prejudice (Fiske et al., 2002; Cary et al., 2017; Vale et al., 2019). Negative depictions of older people (e.g., older people are incompetent) often provoke exclusion (Cuddy et al., 2007) and hostile attitudes (Cary et al., 2017); meanwhile, favorable representations (e.g., older people are warm) incite well-intended benevolent responses, such as giving unnecessary assistance (Cuddy et al., 2005; Cary et al., 2017). Nevertheless, the most common emotional reaction to members who fit the paternalistic stereotype is pity, which inherently has both positive and negative insinuations and highlights the mixed views of older adults (Cuddy et al., 2007). The distinction between benevolent and positive behaviors directed toward older adults can be unclear, as benevolence is often masked as an act of respect or kindness. However, an important division is that benevolence is present when incompetence is inferred and/or the autonomy of older adults is undermined (Cary et al., 2017). In fact, benevolent acts, such as overaccommodative assistance, have been found to be more acceptable when they were directed at an older rather than younger woman (Vale et al., 2019). Therefore, it is important to distinguish these different patterns of ageism, as the great majority of ageism research has a narrow focus on hostile perceptions, despite evidence that benevolent acts of ageism are more commonly and insidiously endorsed (Cherry and Palmore, 2008; Chonody, 2016; Cary et al., 2017; Vale et al., 2019).

The consequences of ageism have grave implications, not only for older adults, but for the rest of the population that will someday advance into late life (Nelson, 2005). According to the stereotype embodiment theory, ageist perceptions are solidified early in life and are internalized, such that they shape self-attitudes, one’s expectations to aging, and ultimately predict health and well-being later in life (Levy, 2009). Much of the work on the impact of ageism supports that positive and negative views of older adults predict cognitive ability, mental health, life expectancy, and likelihood of disease for older adults (Brown et al., 2020; Levy et al., 2020). Additionally, there is support that ageism motivates interactions younger people have with older adults. Ageist attitudes among young adults result in both a higher willingness to give help to older adults, but also a greater likelihood of avoidance and neglect of older adults (Cuddy et al., 2007). Research on ageism among helping professionals (e.g., long-term care workers, nurses, physicians, mental health providers) corroborate that ageism contributes to worse received care for older adults (Robb et al., 2002; Gallagher et al., 2006; Rees et al., 2009; Meisner, 2012). Not only does ageism cause health vulnerabilities and potential mistreatment, it also is a major financial burden with estimated costs of $63 billion dollars per year (Levy et al., 2020).

The COVID-19 pandemic has added to the complexity of attitudes directed toward older adults, because some may blame the dramatic response as an “old people problem,” whereas others may respond with more patronizing behaviors, encouraging vicarious fear and/or pity (Ayalon et al., 2020; Fraser et al., 2020; Lichtenstein, 2020). The popular #*BoomerRemover* tag exemplifies an attitude of defiance in altering pandemic-related health/safety behaviors in order to accommodate the vulnerabilities of Baby Boomers (Fraser et al., 2020; Lichtenstein, 2020). In fact, a recent thematic analysis examining the public’s responses to the pandemic in the United Kingdom, United States, and Australia, noted that use of other ageist epithets, such as *coffin dodger* and *boomer doomer*, are commonly endorsed by younger adults to express hostility toward older adults (Lichtenstein, 2020). Warnings of increased discrimination, neglect, denigration, and amplified devaluing of older adults have also been echoed in pandemic-related commentary (Brooke and Jackson, 2020; Cesari and Proietti, 2020; Morrow-Howell et al., 2020; Petretto and Pili, 2020). Other researchers have supported that older adults are being viewed through a homogenous lens that ignores the vast diversity within this group (Ayalon, 2020; Ayalon et al., 2020). Assimilated perceptions of older adults as a vulnerable group likely reinforces paternalistic perceptions that infer older adults are fragile and vulnerable, especially amidst the pandemic (Lichtenstein, 2020; Rahman and Jahan, 2020). In fact, many health agencies (e.g., long-term care) and individuals (e.g., adult children) have responded to the pandemic in benevolent manners with the intention to protect older adults; however, overaccommodative polices and/or behaviors could be harmful for older adults as they may undermine older adults’ social and emotional health, autonomy, and their right to make their own health-based decisions (Ayalon, 2020; Lichtenstein, 2020; Rahman and Jahan, 2020). For example, sequestering older adults, or avoiding contact with older adult family members, risks increasing social isolation which can ultimately have negative ramifications for health (Ayalon, 2020; Lichtenstein, 2020; Morrow-Howell et al., 2020). Comprehensively, attitudes toward older adults are embedded in the social context surrounding the salience of the pandemic, and these attitudes may play a role in predicting how people incorporate protective safety and hygiene behaviors.

Understanding the process of behavior change in order to create beneficial interventions has been a key goal for social and medical scientists (Ajzen, 1991, 2015). The theory of planned behavior (TPB) is one of the most commonly used theories of successful behavior change and posits that unique motivators, based on an individual’s beliefs, precipitate behavioral intensions that ultimately predict an anticipated action (Ajzen, 1991, 2011, 2012). Behavioral intentions involve different belief aspects motivating one’s readiness to complete the desired behavior, and include, but are not limited to, attitudes toward the behavior, subjective norms, and factors of perceived control (Ajzen, 1991). For example, the TPB specifies that general attitudes, such as ageism, connect to behavior via contextualized precursory intentions. Since its conception, countless applications of the TPB have been used to examine how to modify behaviors, such as learning how to reduce risky behaviors (e.g., smoking) or promoting social interactions (Ajzen, 2011, 2012). In particular, the TPB framework has guided efforts delineating how attitudes toward older adults predict younger adults’ intentions to engage with older adults and/or promote intergenerational relationships (Bousfield and Hutchison, 2010; Joshi et al., 2015; Reuveni and Werner, 2015). Although these studies do support that attitudes toward aging are antecedents in predicting behavior, they provide a very narrow focus and could be improved by incorporating benevolent in addition to hostile attitudes, exploring older adults’ ageist perceptions given that they are just as likely to endorse ageist attitudes as younger adults (Cherry and Palmore, 2008; Levy, 2009), and connecting ageism to other behaviors that influence health, well-being, and treatment of older adults. Given that the TPB is a useful framework utilized by interventionists and professionals to promote positive behavioral change, it is suitable to integrate the TPB into the current project as it will help outline the process of whether ageism nurtures or inhibits positive responses to the COVID-19 pandemic. Delineating these links would assist professionals attempting to promote compliance with COVID-19-related health and safety regulations in addition to those wishing to underscore the relevance of deleterious ageist ideology.

**FIGURE 1:**
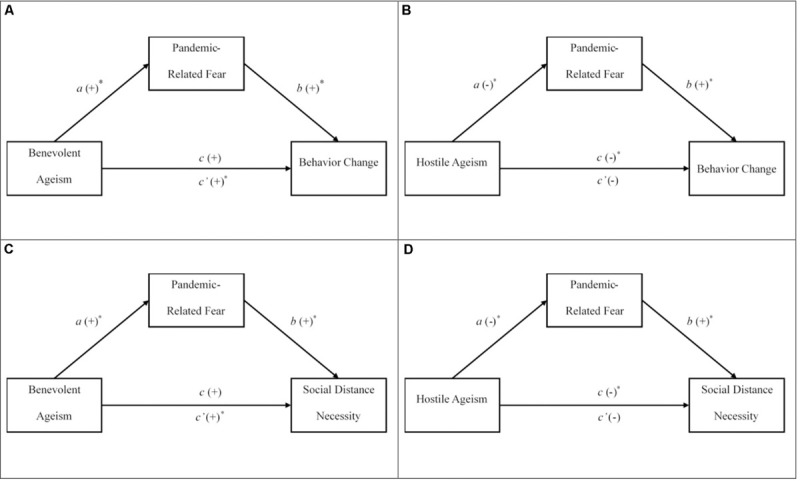
Hypothesized mediation models assessing the indirect relationships between benevolent ageism and responses to the pandemic. The panels are graphical representations of the expected path models for hypotheses 3 **(A,B)** and 4 **(C,D)**. The direct effect is represented by *c* and the indirect effect is represented by *c*′. ^∗^path is expected to be significant at *p* < 0.05.

The rise of ageism during the COVID-19 pandemic provides an optimal opportunity for researchers to extend work on ageist attitudes to salient health-related behavior modifications that could not be created in artificial research scenarios (Ayalon et al., 2020; Lichtenstein, 2020). The pandemic presents challenges for individuals’ internalization of ageist ideology, and additionally provides unique health implications to the lives of older adults (Morrow-Howell et al., 2020). The primary goal of the project was to examine if benevolent and hostile ageism predicted different responses to the COVID-19 pandemic such as: pandemic-related fear, self-reported pandemic-related behavior change, and the necessity of social distancing. In this study, pandemic-related fear refers to how concerned individuals feel about the pandemic in terms of their own and loved one’s safety. Pandemic-related behavior change refers to self-reported experiences of how people have altered their behavior since the start of the COVID-19 pandemic^1^. Lastly, the perceived necessity of social distancing is examined specifically, given that this was the most crucial protective factor recommended by the Center for Disease Control (2020) at the time of study development. The exploratory goal of the study was to explore how benevolent and hostile attitudes lead to different patterns in responses to the pandemic (i.e., fear, behavior changes, perceptions toward social distancing) using the general framework suggested by the TPB. More specifically, we expected that pandemic-related fear, would constitute as a behavioral intention that would mediate the link between ageism and pandemic-related behavior change and the overall perceived necessity of social distancing, respectively (see Figure 1 for illustrations). Hypotheses 1 and 2, specifically referring to pandemic-related fear and behavior change, were preregistered and the remaining hypotheses were exploratory^2^.

Given that that maintaining the health and safety of older adults has been used to justify the dramatic responses to the pandemic, those who are higher in hostile ageism will be more likely to view the pandemic as an ‘older person’s’ disease and thus will be less fearful of the pandemic and make less effort to adapt their behavior. However, the negative links between hostile ageism and pandemic-related behavior change and the perceived necessity for social distancing will not be mediated through pandemic related fear, because these negative associations will not raise concern to motivate adaptive responses. On the other hand, those high in benevolent ageism will have more concern for the safety of older adults, as they will view them more paternalistically, and thus will respond with more fear and will alter their behavior accordingly (see Figure 1 for illustrations of our expected results).

*Hypothesis 1*: Higher endorsement of hostile ageism will predict lower fear related to the COVID-19 pandemic, less self-reported behavior change related to the COVID-19 pandemic, and lower perceived social distancing necessity.
*Hypothesis 2*: Higher endorsement of benevolent ageism will predict higher fear related to the COVID-19 pandemic, more self-reported behavioral change related to the COVID-19 pandemic, and higher perceived social distancing necessity.
*Hypothesis 3*: The positive link between benevolent ageism and self-reported changes in behaviors will be mediated through fear related to the COVID-19 pandemic, such that benevolent ageism will positively predict higher pandemic-related fear which will positively predict more pandemic-related behavior change; however, the negative relation between hostile ageism and pandemic-related behavior change will not be mediated through pandemic-related fear.
*Hypothesis 4*: The positive link between benevolent ageism and perceived higher social distancing necessity will be mediated through fear related to the COVID-19 pandemic, such that increases in benevolent ageism will predict higher pandemic-related fear which will positively predict higher social distance necessity; however, the negative relation between hostile ageism and social distancing necessity will not be mediated through pandemic-related fear.

## Materials and Methods

### Participants

Initially, data from 363 participants were collected online as part of a larger preregistered project examining age differences in thoughts, feeling, and behaviors related to the COVID-19 pandemic; however, 28 (∼8%) respondents were removed from the study because: their responses were invalid (*n* = 2), they did not meet inclusion age criteria (*n* = 22), or they provided less than 10% complete data (*n* = 4). Responses from 164 younger (ages 18–30) and 171 older adults (ages 60–80) were retained. Age differences were not of focal interest to this study; therefore, the two groups were combined, and age group was considered a covariate in data analysis. More information regarding age differences are presented in the Supplementary Materials. The sample was primarily female (73.40%) compared to being male (26.00%) or gender fluid (<1%), and predominantly White (91.3%) compared to other races (i.e., Black, Asian, Mixed Race, American Indian/Alaskan Native). The sample held high levels of education, with few selecting their highest level of education as completing high school (10.40%), many participants had some college (30.70%), more held at least a Bachelor’s degrees and/or some graduate school experience (34.00%), and lastly a quarter of the sample held a graduate school degree or higher (24.80%). Further, about half the sample identified their political affiliation as primarily democrat (47.50%), with 24.20% identifying as republican, 24.80% as independent and 3.60% indicating another political affiliation that was not listed. Lastly, a quarter of the sample (25.10%) reported being an essential worker.

### Power Analysis

Sample size was determined using a Monte Carlo power analysis simulation for testing indirect effects for mediation with bootstrapped confidence intervals that revealed a minimum sample size of 300 (Schoemann et al., 2017). The simulation determined the sample size necessary to detect significant indirect effects for mediation using 10,000 bootstrapped distributions with 95% confidence intervals (CI) adjusted for industry standard error rate (α = 0.05), statistical power (β−1 = 0.80), and small effect sizes (*r* = 0.20) across all pathways involved in the mediation models. Given the novelty of this line of inquiry we used small effect sizes to be conservative in our estimation.

### Design and Procedure

Participants were asked to respond to an online survey that took approximately 20–30 min. to complete. In order to participate, individuals had to be between the ages of 18–30 or 60–80 years old, had to be a native English speaker, and live in the United States. The online study was compiled of a series of different questions assessing thoughts and behaviors directed toward the pandemic, several established psychosocial questionnaires, and other interactive tasks outside the scope of the current endeavor (see preregistered website for more details^3^). Participants were recruited with social media postings embedded with links to the survey, recruitment by email from an existing older adult participant database, and through Amazon Mechanical Turk Panels using TurkPrime.com (Litman et al., 2017). Turk participants were recruited through Turk Panels that verify participant background characteristics, given our age-specific recruitment needs. All participants had to pass attention checks throughout the survey to be included. No monetary incentive was provided to those recruited through social media. To prevent selection bias of those most willing to participate, we also recruited subjects from Amazon Mechanical Turk (MTurk), who did receive a small monetary compensation after completion of the study (34.60% of the sample were recruited on MTurk). Nearly half of the sample were residents of the state of Ohio (51.30%) and the other half lived in 38 other states. Those recruited through social media were mostly made up of residents from Ohio (76.70%), which was significantly more than the number of individuals from Ohio recruited through MTurk, χ^2^(1) = 162.93, *p* < 0.001. The social media recruitment was completed between April 26th and May 9th and the MTurk data were completed on May 3rd and 4th, nearing the 100-day mark of the first confirmed case of the COVID-19 virus in the United States (Holshue et al., 2020). Data collection occurred during the *Stay Safe Order* in Ohio, extending the ban of mass gatherings and stay at home orders that were issued roughly a month before and was prior to the reopening of the state for non-essential services (Ohio Department of Health, 2020). Similar orders were also issued in 32 other states, though the nuances and lengths of the orders varied greatly (National Academy for State Health Policy, 2020). During this period, the number of cumulative cases of the COVID-19 virus in the United States ranged from 830,053 to 1,245,874 (Statista, 2020).

### Measures

#### Ageism

The 13-item Ambivalent Ageism Scale was used to measure positive and negative attitudes directed toward older adults (Cary et al., 2017). The 9-item Benevolent Ageism subscale examines the more insidious aspect of ageism and highlights aspects of ageism that are seemingly positive but assume older adults to be incompetent (α = 0.90). Example items of benevolent ageism include, *“Older people need to be protected from the harsh realities of society”* and *“Even though they do not ask for help, older people should always be offered help.”* The 4-item Hostile Ageism subscale assesses the negative perception of older adults (α = 0.90). Example items of hostile ageism include, *“Old people are a drain on the healthcare system and economy”* and *“Old people are too easily offended.”* Both subscales were measured on a scale of 1 (*Strongly Disagree*) to 7 (*Strongly Agree*).

#### Pandemic-Related Fear

Fear of COVID-19 (i.e., coronavirus) was assessed with a composite score of 4 items created for this study. The items were, *“How afraid are you of contracting the coronavirus?”*, *“How often in the last week did you fear that you would contract the coronavirus?”*, *“How often in the past week did you fear one of your loved ones would contract the coronavirus?”*, and *“How often in the last week did you think about the coronavirus?”* The first item was scaled between 1 (*Not at All*) and 10 (*Extremely*), and the remaining items were scaled from 1 (*Not at All*) to 10 (*Extremely Often*). A composite score was created by averaging the 4 items so that scores ranged between 1–10 and exhibited acceptable reliability (α = 0.80).

#### Pandemic-Related Behavior Change

Changes in behaviors due to the coronavirus were assessed by having participants rate how they have changed certain hygiene and safety-related habits since the outbreak began in the United States. The six safety-related habits were: frequency of washing hands, duration of washing hands, frequency of visiting stores, amount of time spent inside stores, and frequency of leaving their house/property, and were examined on a scale of 1 (*Extremely Decreased*) to 9 (*Extremely Increased*)^4^. The last four items were reverse-scored so that higher scores indicated more safety-related behavior change due to the coronavirus. A composite behavior change score was created by averaging the six items, which exhibited acceptable reliability (α = 0.75).

#### Social Distancing Necessity

A single item was used to assess agreement with the importance of social distancing. More specifically, participants were asked to rate their agreement to the following question, *“What are your feelings about the necessity for social distancing?”*, on a scale of 0 (*Disagree Completely*) to 100 (*Completely Agree*).

## Results

### Data Preparation

Prior to data analysis, missing data, normality of the data, and potential outliers were examined. There was an 11% attrition rate of participants who dropped out before completing the survey, likely due to fatigue and/or being distracted by other online activity, and they were dropped from the current study. There were no significant differences on the focal constructs between those who finished and those who dropped out of the survey. Only two participants that remained in the sample had incomplete data, therefore, data from all available participants were retained for each analysis. Normality was examined using kurtosis and skewness statistics on SPSS and the use of histograms for each variable (Osborne, 2002; Tabachnick and Fidell, 2007). The fear variable appeared to be normal, both ageism metrics were positively skewed, behavior change was negatively skewed, and social distancing necessity appeared to be severely negatively skewed and kurtotic. The lack of normality in the ageism variables were corrected with log10 data transformations and a square root data transformation was used for the behavior change variable (Osborne, 2002). When rating social distancing necessity, roughly half (50.7%) of the sample rated that they “completely agreed” and had a rating of 100. In order to accommodate the lack of distribution of scores and address the skewness, the variable was recoded so that scores between 0–20, 21–40, 41–60, 61–80, 81–99, and those that chose 100 were grouped together. The rescaled variable was very highly correlated with the original metric (*r* = 0.97) and the rescaling did improve the normality of the distribution, but there was still a negative skew and slight kurtosis that was not improved with further data transformations. The raw rescaled social distancing variable was retained and is interpreted with caution^5^. Univariate outliers and multivariate outliers were examined using p-plots, transforming scores into standardized *z* distribution, and Mahalanobis distance, respectively (Tabachnick and Fidell, 2007). There were no noticeable univariate or multivariate outliers when examining the transformed and/or rescaled data.

### Descriptive Statistics and Control Variables

The means, standard deviations, and interrelationships among the focal constructs are presented in Table 1. These metrics are also presented by age group in Supplementary Table 1. The sample had relatively low ageism scores, but these were equitable to the means in the original Ambivalent Ageism Scale validation study (Cary et al., 2017). The sample rated average mean levels of fear, high behavioral change, and strongly agreed with social distancing practices. The ageism subscale scores had a stronger positive correlation than in the original validation scale, *r*(159) = 0.62 (Cary et al., 2017). Fear was significantly correlated with both behavioral change and social distancing necessity, and both were significantly correlated with each other. It appeared that the young adult group was driving the relationships between the ageism variable and the responses to the pandemic; however, age group did not moderate any of these links, suggesting that younger and older adults had similar relationships between ageism and responses to the pandemic (the results of these moderations can be found in Supplementary Table 2).

**TABLE 1:** Descriptive statistics and correlations for ageism and pandemic-related responses.

**Variable**	**1**	**2**	**3**	**4**	**5**
(1) Benevolent ageism	–				
(2) Hostile ageism	0.72**	–			
(3) Pandemic related fear	0.13*	0.10^†^	–		
(4) Behavior change	−0.24**	−24**	0.22**	–	
(5) Social distance necessity	−0.18**	−0.15**	0.28**	0.41**	–
*M*	2.47	2.38	5.56	7.56	87.07
*SD*	1.21	1.22	2.09	1.09	21.52
*N*	333	333	335	335	335

Transformed or rescaled data were used in the correlation matrix, and raw data were used for the means and standard deviations. The mean and standard deviation of the rescaled social distancing variable were 5.07 and 1.26, respectively. Two participants dropped out of the survey before the Ambivalent Ageism Scale was administered but were maintained in all other analyses. *p < 0.05, **p < 0.01, ^†^p < 0.10.

Differences among the focal constructs were explored across multiple participant background characteristics that have been noted to influence reactions the COVID-19 pandemic (Barber and Kim, 2020; Campos-Castillo, 2020; Garcia et al., 2020; Gauthier et al., 2020). These factors included age group (younger adult vs. older adult), gender (male vs. female), race (White vs. non-White), level of education, recruitment source (social media vs. MTurk), state of residency (Ohio vs. other), political affiliation, and essential worker status. There were no significant differences between men and women among any focal constructs, but age group, political affiliation, race, recruitment source, state of residency, essential worker status, and level of education were all considered covariates because they differed on at least one focal construct. More specifically, higher ageism scores were found for younger, non-White, less educated, republican and independent, non-Ohio residing, and MTurk-recruited participants. Political affiliation was the only background factor related to pandemic-related fear, with democrats reporting more fear than the other groups. Higher scores of pandemic-related behavior change were found in older, democrat, highly educated, and non-essential working participants. Lastly, social distancing necessity was higher among older, democrat, and non-essential working participants. All covariates were controlled for in mediation models, and political affiliation and level of education were dummy coded with democrats and those with the most education serving as the comparison groups.

### Analytic Plan

Given the novelty and lack of research on the topic, we used basic bivariate correlations to test hypotheses 1 and 2, which are listed in Table 1. The goal of these basic correlation tests was to confirm the associations among the constructs. Hypotheses 3 and 4 were tested with regression-based mediation using Fiske et al. (2002) PROCESS macro for SPSS. More specifically, Model 4 with 10,000 bootstrapped samples was used for both hypotheses. Given that the mediations build off the results from hypotheses 1 and 2, they also further establish support for the hypothesized relationships. Mediation analyses require the analysis of two multiple regressions. The first regression tests how the antecedent, beyond control variables, predicts the mediator (i.e., *a*-path). The second regression tests how the mediator, beyond the antecedent and control variables, predicts the criterion (i.e., *b*-path; see Figure 1). In essence, the *a*-path model is a regression testing how ageism predicts fear associated with the pandemic and is identical across our hypothesized mediation models. Because there are two different criterion outcomes, there are two *b*-path regression analyses exploring how pandemic-related, fear, and background factors uniquely predict pandemic-related behavior change and social distancing necessity, respectively. Parameter estimates for the *a*- and *b*-path regressions can be found in Table 2. The advantages of using the PROCESS methodology are that the program quantifies the indirect effect (*ab*), corrects for non-normality of the indirect effect with bootstrapping, and provides confidence intervals around parameter estimates (Hayes, 2013). Therefore, untransformed data for the ageism and behavioral change variables were used, as bootstrapping corrects for normality violations. If confidence intervals do not include zero, they are interpreted as being significant, and support for mediation is indicated with a significant indirect effect and a non-significant direct effect.

**TABLE 2:** Regression coefficients for mediational path models.

**Variable**	**Pandemic-related fear**			**Pandemic-related behavior change**			**Social distance necessity**		
	***b***	***se***	**95% CI**	***b***	***se***	**95% CI**	***b***	***se***	**95% CI**
Republican	−1.61**	0.29	[−2.19, −1.04]	−0.211	0.15	[−0.51, 0.09]	−0.47**	−0.18	[−0.82, −0.11]
Independent	−1.13**	0.27	[−1.67, −0.59]	−0.19	0.14	[−0.47, 0.08]	−0.31†	0.16	[−0.63, 0.02]
Other political affiliation	−1.19*	0.61	[−2.38, −0.001]	0.08	0.30	[−0.51, 0.67]	0.15	0.36	[−0.55, 0.85]
Bachelor’s degree	−0.19	0.30	[−0.78, 0.39]	−0.10	0.15	[−0.38, 0.19]	0.13	0.17	[−0.21, 0.48]
Some college	0.11	0.32	[−0.52, 0.74]	−0.23	0.16	[−0.53, 0.08]	0.04	0.19	[−0.33, 40]
Some high school	0.36	0.42	[−0.47, 1.19]	0.01	0.21	[−0.40, 0.42]	0.06	0.25	[−0.42, 0.55]
Age group	0.001	0.25	[−0.49, 0.50]	0.33**	0.12	[0.08, 0.57]	0.51**	0.15	[0.22, 0.80]
Recruitment method	0.72*	0.35	[0.04, 1.40]	−0.27	0.17	[−0.61, 0.07]	−0.30	0.20	[−0.70, 0.10]
State	0.36	0.31	[−0.25, 0.97]	−0.13	0.15	[−0.44, 0.17]	−0.09	0.18	[−0.45, 0.26]
Race	0.67	0.41	[−0.13, 1.47]	−0.26	0.20	[−0.65, 0.14]	0.03	0.24	[−0.44, 0.50]
Essential worker	−72**	0.27	[−1.24, −0.19]	0.40**	0.13	[0.13, 0.66]	0.29	0.16	[−0.02, 0.60]
Benevolent ageism	0.36**	1.21	[1.55, 6.43]	−0.11	0.07	[−0.25, 0.03]	−0.02	0.08	[−0.19, 0.14]
Hostile ageism	−0.06	0.13	[−0.31, 0.20]	−0.12	0.06	[−0.25, 0.002]	−0.07	0.08	[−0.22, 0.08]
Pandemic-related fear	–	–	–	0.13**	0.03	[0.08, 0.19]	0.18**	0.03	[0.12, 0.25]

N = 233 across all models. Political affiliation and education were dummy coded due to their categorical nature. The reference group for political affiliation was democrat and the reference group for education level was those who hold a graduate degree or higher. The following coding schemes were used for age group (1 = younger adult, 2 = older adult), recruitment method (1 = social media, 2 = MTurk), state (1 = not from Ohio, 2 = Ohio), essential worker status (1 = essential worker, 2 = non-essential worker), and race (1 = Non-White and 2 = White). Raw data for the focal constructs were used as bootstrapping corrects for normality violations. The first model predicating pandemic-related fear represents the results for the a-path models across all the mediation analyses. The second regression predicting pandemic-related behavior change represents the b-path models for the mediations testing hypothesis 3, and the last regression predicting social distance necessity represents the b-path models for the mediations testing hypothesis 4. *p < 0.05, **p < 0.01, ^†^p < 0.10.

### Preregistered Hypotheses

The first two hypotheses suggesting that lower hostile ageism and higher benevolent ageism would predict pandemic-related fear were partially supported (see Table 1 for full correlation matrix). Higher scores of benevolent ageism were significantly and positively correlated with fear (*r* = 0.13, *p* = 0.02). Higher hostile ageism was positively related to fear; however, this relationship was not statistically significant (*r* = 0.10, *p* = 0.07). Further, both ageism predictors significantly correlated with behavioral change (*r* = −0.24, *p* < 0.001; *r* = −0.24, *p* < 0.001, respectively). Contrary to expectation, benevolent ageism was negatively correlated with behavioral change.

### Exploratory Hypotheses

**FIGURE 2:**
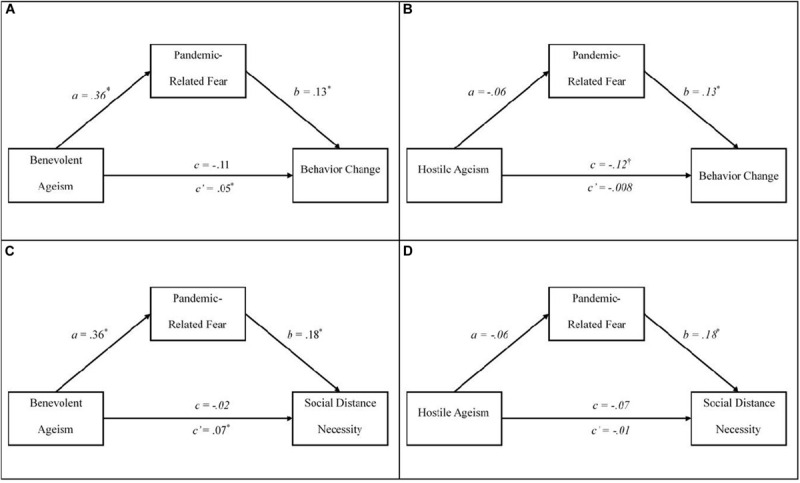
Mediational models examining the indirect relationships between ageism and responses to the pandemic. Each panel represents our unique mediation models for hypotheses 3 **(A,B)** and 4 **(C,D)**. *N* = 233 across all models. The direct effect is represented by *c* and the indirect effect is represented by *c*′. Each analysis controlled for the political affiliation, age group, recruitment method, participant race, participant home state, essential worker status, degree of education, and the other respective type of ageism. Table 2 provides the parameter estimates for these background factors. ^∗^*p* < 0.05.

The first exploratory analysis was to examine if lower hostile and higher benevolent ageism predict higher agreement with social distancing necessity. Both ageism facets significantly predicted social distance agreement (*r* = −0.15, *p* = 0.005; *r* = −0.18, *p* = 0.001, for hostile and benevolent ageism, respectively), but benevolent ageism was correlated in the opposite direction to that hypothesized. The final two hypotheses explore the application of the TPB and outline that benevolent, but not hostile, ageism would predict responses to the pandemic (i.e., pandemic-related behavior change, perceived social distancing necessity) through pandemic-related fear. It was expected that benevolent ageism would predict more fear (i.e., the *a-*path), which in turn would predict more adaptive pandemic responses (i.e., the *b-*path; see Figure 2 for detailed depiction).

Two mediation models, one with a benevolent and one with hostile ageism as the antecedent, were run to test hypotheses 3 and 4. The list of aforementioned covariates and the other ageism facet were included in these models; therefore, the parameter estimates across the two models were identical with the only difference being the calculation of the indirect and direct effects (see Table 2 for parameter estimates for all variables). Figure 2 illustrates the results for each mediational model. Hypothesis 3, exploring if pandemic-related fear mediates the relation between benevolent ageism and behavior change was supported (see Figure 2A), with the indirect effect being significant, *B* = 0.05, *se* = 0.02, 95% CI [0.01, 0.10], and the direct effect not significant, *B* = −0.11, *se* = 0.07, 95% CI [−0.25, 0.03]. Unexpectedly, the indirect and direct effects were in opposite directions, indicating inconsistent mediation or suppression rather than typical mediation (MacKinnon et al., 2000; Hayes, 2013). Mediators and suppressor variables are both types of third variable effects (MacKinnon et al., 2000). A suppressor variable increases the relationship between other variables when it is included in the equation, whereas a mediator reduces the direct effect between two variables (MacKinnon et al., 2000). The finding of a suppressor effect is not surprising given the relationship between benevolent ageism and behavior change were in the opposite expected direction, highlighting the inconsistency in the hypothesized model (i.e., hypothesis 2). Nonetheless, the *a*-path, of benevolent ageism predicting pandemic-related fear was consistent with expectations, *B* = 0.36, *se* = 0.14, 95% CI [0.08, 0.63], even when controlling for hostile ageism and other covariates. The *b*-path, pandemic-related fear predicting behavior change, was consistent with expectations and significant when controlling for both facets of ageism and covariates, *B* = 0.13, *se* = 0.03, 95% CI [0.08, 0.19]. However, there was no evidence of mediation or suppression when hostile ageism was the antecedent (see Figure 2B), as the indirect, *B* = −0.01, *se* = 0.02, 95% CI [−0.04, 0.02], and the direct effects, *B* = −0.12, *se* = 0.06, 95% CI [−0.26, 0.002], were not significant. It should be noted that the *a*-path was not supported in this model, indicating that hostile ageism was not a significant predictor of fear when the covariates and benevolent ageism were also included. There was support for hypothesis 3 and the TPB: benevolent ageism does increase fear, which in turn increases behavior change. On the other hand, hostile ageism did not predict behavior change, when controlling for covariates, fear, and benevolent ageism.

When the agreement with social distancing necessity was used as the outcome for hypothesis 4, the same pattern emerged between benevolent and hostile ageism. Pandemic-related fear acted as a suppressor and increased benevolent ageism’s relationship with social distance necessity (see Figure 2C), as there was a significant indirect effect, *B* = 0.07, *se* = 0.03, 95% CI [0.02, 0.13], and a non-significant direct effect in the opposite direction, *B* = −0.02, *se* = 0.08, 95% CI [−0.19, 0.14]. The *a*-path was identical to hypothesis 3 and was significant. The *b*-path of pandemic-related fear predicting social distance necessity was significant, *B* = 0.18, *se* = 0.03, 95% CI [0.12, 0.25]. When hostile ageism was the antecedent (see Figure 2D), neither the direct, *B* = −0.07, *se* = 0.08, 95% CI [−0.22, 0.08], nor the indirect effect were significant, *B* = −0.01, *se* = 0.02, 95% CI [−0.06, 0.03]. In sum, we supported hypotheses 3 and 4, as benevolent ageism predicted fear, or the intention to change, which then predicted behavior change and social distance necessity importance, respectively.

## Discussion

The COVID-19 pandemic has reshaped ageism in the United States and will have lasting implications for older adults (Morrow-Howell et al., 2020). The findings from this study confirm that ageist attitudes predicted responses to the COVID-19 pandemic for both younger and older adults. Hostile attitudes correlated with perceiving less necessity for social distancing and did not predict pandemic-related behavior change, when controlling for numerous background factors (e.g., age group, political ideology, essential worker status) and benevolent ageism. Associations with benevolent ageism initially mirrored those with hostile ageism showing negative correlations with behavior change and social distancing necessity; however, these relationships reversed in direction once pandemic-related fear was considered. Pandemic-related fear served as a suppressor variable, rather than a mediator, such that higher scores of benevolent ageism were associated with more fear, and higher fear predicted positive behavior change and increased social distancing necessity, respectively. Overall, each hypothesis was at least partially supported, with the only exception being that in hypothesis 1, hostile ageism did not relate to pandemic-related fear.

The current findings benefit the study of ageism and the scientific understanding of promoting safety and hygiene (e.g., health practices like hand washing) during a global pandemic. Hostile and benevolent ageism had divergent patterns in predicting responses to the pandemic indicating the complexities of assessing attitudes toward older people and warranting that researchers and professionals must consider both in order to have a complete grasp on the nature of ageism. Professionals developing public messages around older people or working directly with this group during the pandemic should consider their own ageist biases, and the biases of their intended audience, as all people fall in different places on the spectrum of ageism and their attitudes might influence how they interpret public messages. For example, a person high in hostile ageism might respond to such public messaging with less intention to change their behaviors with efforts that aim to protect older adults. In contrast, an individual higher in benevolent ageism might interpret a similar public message in an overzealous manner, which could result in paternalistic or belittling behaviors toward older adults. Further, it is important that professionals keep in mind how they represent older people in the context of the pandemic, as these messages could have indirect consequences on how older people are viewed and thus treated, which ultimately can influence their health (Ayalon, 2020; Ayalon et al., 2020; Lichtenstein, 2020; Morrow-Howell et al., 2020).

The findings of this study may also add to prior work indicating that hostile attitudes can compromise the quality of care provided by healthcare workers such as nurses (Rees et al., 2009), long-term care workers (Gallagher et al., 2006), doctors (Meisner, 2012), counselors/therapists (Robb et al., 2002), and even older adult patients who internalize hostile beliefs (Makris et al., 2015). More specifically, this study generalizes established findings on hostile ageism to a new paradigm concentrating on more subtle health and safety behaviors provided by non-professionals. Additionally, the innovative application of benevolent ageism, based on the general TPB framework, integrates the relevance and presence of the construct beyond young adults’ attitudes toward older people. More development is needed to fully assess the harm and/or benefit of benevolent ageism as the construct demonstrated complex patterns with the suppression effect changing the directionality between benevolent ageism and responses to the pandemic when fear was considered. While the finding that those high in benevolence predicted more behavior change could be concluded as a positive outcome, this effect functions as a result of increased fear and may covertly reinforce deep-seated attitudes that older adults are incompetent. Very little is known about benevolent ageism from the perspective of older adults, including potential correlates with health and how it is even perceived (Chasteen et al., 2020). Indirectly undermining older adults’ feelings of competence could be harmful, as feelings of competence are known predictors of well-being in very old age (Neubauer et al., 2017). Overall, these findings integrate with and build upon the literature and conceptualization of ageism, and are especially important considering ageism is costly, related to numerous health vulnerabilities in older adulthood, and will someday be relevant for persons of all ages (Levy et al., 2020).

One of the greatest challenges for health specialists and public figures throughout the pandemic has been to promote and maintain precautionary safety and health measures proposed by the Centers for Disease Control and Prevention (Center for Disease Control, 2020). The imposition of these safety requirements has resulted in increased stress and backlash among people in the United States (American Psychological Association, 2020). The current study underscores that underlying hostile attitudes toward older people may curtail the salience and gravity of such lifestyle responses to the pandemic. In other words, those high in hostile ageism might not take the disease as seriously due to its prominent association with older people. On the contrary, those high in benevolent ageism might not feel these responses are relevant to their own health and safety, as evidenced with benevolent ageism’s initial negative association with an individual’s behavior change, but rather make changes to protect and assist older adults as the direction between the two switched when pandemic-related fear (i.e., for self and others) was modeled with these factors (see Figures 2A,C). Although older adults are at higher risk for COVID-19 related mortality, trends at the time of this writing suggest that there are spikes in the confirmed cases of the disease in young adults. This suggests that younger people might be viewing the pandemic as an issue for older people and be less inclined to take precautionary action in order to attenuate the spread of the disease (Bosman and Mervosh, 2020). Older adults who internalize hostile ageism might be especially susceptible in such cases, as they may not be taking their health vulnerabilities earnestly, and consequently make less changes in behavior. Conversely, older adults who internalize benevolent ageism might take their health vulnerabilities too seriously and demoralize their own abilities and minimize their perceived sense of control, which is a definitive feature of predicting well-being in late life (Neubauer et al., 2017). It is vital that those interacting with older adults (e.g., professionals, family members, and neighbors) consider their own ageist assumptions in relation to how older adults should deal with the pandemic (Ayalon et al., 2020). For example, perceiving older adults as defenseless to the disease and/or minimizing their abilities to make informed decisions regarding safety precautions may reward benevolent attitudes and diminish feelings of self-efficacy and competence, and ultimately have grave implications for well-being (Neubauer et al., 2017).

While the nature of this study relies on unique experiences during the pandemic, the implications from these findings generalize beyond this limited scope to other everyday contexts where older adult stereotypes influence behavior. Some examples include: having less willingness to have social interactions with older people (Cadieux et al., 2019), giving poorer service to older adults (Chasteen et al., 2020), recognizing symptoms of depression as normal signs of aging (Smith and Meeks, 2019), making assumptions of physical abilities of older adults and offering unwanted help (Vale et al., 2019), use of patronizing speech that assumes cognitive decline (Kemper, 1994), and even feelings of endearment enacted because of pity (Cuddy et al., 2005; Cherry and Palmore, 2008). These everyday behaviors are directed toward older adults, but little is known about the consequences they have for older adults and how they are preceded by the individual’s own ageist attitudes. Consideration of the safety and vulnerability of older adults extends beyond COVID-19, and these results highlight the importance of considering the role of ageist assumptions on health-related behavior change.

### Limitations and Future Directions

As with any study, the current project is not without limitations. Although the sample was adequately powered, it was relatively small, and could be biased due to high responses from liberal and well-educated participants. Similarly, most participants were from Ohio, which may limit the generalizability of the findings given that different geographic regions varied in their responses to the pandemic (National Academy for State Health Policy, 2020). The small effect sizes from correlations and suppression effects are unsurprising, as general attitudes historically do not have strong relationships with specific behaviors (Ajzen, 2012). However, these connections are a first step toward developing a more complete understanding of how ageism influences behavior change. The cross-sectional and correlational aspects of the study design also limit potential inferences made from the data. More specifically, these findings do not apply to concrete predicted behavior change, but rather retrospective self-reported accounts. And the mediation/suppression cannot indicate causality due to potentially bidirectional correlational links and lack of temporal precedence. The directionality of the effects is supported by the general theoretical TPB framework, but the current study did not fully encapsulate all aspects of the framework. More specifically, behavioral intentions were not fully measured and instead were assumed to be indexed by pandemic-related fear which could be more of a precursor belief factor rather than a behavioral intention. The integration of examining how ageism can be applied in a TPB context can be improved by incorporating behavioral intentions and other factors such as feelings of control and social norms. Lastly, the scope and measurement of the focal constructs could have been improved. Specifically, other important pandemic-related changes in behaviors such as the wearing of masks and other equipment, the amount of touching one’s face, and the amount of sanitization were not examined. Similarly, we could have expanded our conceptualization of pandemic-related fear to be more inclusive of actual concern of the pandemic, feelings of fear felt on behalf of older adults, and feelings of pity directed at older adults. These approaches would have offered more direct ways to capture concern for the pandemic, rather than relying on how individuals felt for their own welfare. The lack of variability issue with the social distancing necessity question could have also been improved by adding more thorough and inclusive questions. Nonetheless, these data do provide a unique snapshot of how attitudes influence health-related behavior change in a pandemic. The findings are also subject to history effects given that data were collected within a constantly changing context during the pandemic. For example, social distancing necessity may have been perceived to be more necessary at the start of the pandemic when we collected data, and diminished as time passed and safety and regulation trends developed. While our models could be improved, they still provide important novel findings that move research on ageism and the understanding of the response to the pandemic forward.

Future researchers and professionals addressing the lives of older adults need to extend our concepts of ageism, especially in times when older adult health and safety is uniquely relevant. Hostile ageism is undoubtedly associated with negative outcomes, but ageism research needs to disentangle the harm of benevolent ageism both in how it directs behaviors toward older adults, and how it is received by them. Future researchers should capitalize on theoretical and methodological infrastructure provided in the TPB to extend knowledge on how ageism influences other everyday behaviors. Moreover, integration of this theory would build a better understanding of how to target ageism interventions (Burnes et al., 2019). As a response to the pandemic, the Gerontology Society of America (2020) released an *Ageism First Aid* multimodule course to recognize ageism in the health and helping professions. Other researchers also have recently attempted to demonstrate that ageism can be attenuated through educational interventions, intergenerational contact, and correcting the paternalistic stereotypes (Levy, 2018; Burnes et al., 2019; Cadieux et al., 2019; Vale et al., 2019). Less is known on how to implement these findings on a larger scale, but these programs and efforts are needed, especially given that the responses to this pandemic have reinforced traditional ageist ideology. Ageism and attitudes toward older adults have been reshaped by the pandemic and future research and intervention is needed to understand the extent to which ageist attitudes inform important behavioral decisions.

## Conclusion

The COVID-19 pandemic has presented a unique rise in the salience of ageism in America. Maintaining the safety of older adults, and other vulnerable groups has been used to justify necessary behavior changes that have restructured the lives of many Americans. The findings of this preregistered study confirm that hostile ageism was associated with less pandemic-related health and safety precautions and that benevolent ageism related to increased behavior changes, but only as a result of increased pandemic-related fear. These timely findings suggest that those working on addressing pandemic-related health issues must consider ageist assumptions as they may predict how we deal with such circumstances.

## Data Availability Statement

The datasets generated for this study can be found in online repositories. The names of the repository/repositories and accession number(s) can be found below: https://osf.io/ynbm3.

## Ethics Statement

The studies involving human participants were reviewed and approved by University of Akron Institutional Review Board (IRB# 20200403). The patients/participants provided their informed consent to participate in this study.

## Author Contributions

MV, JS, MH, AV, and JT contributed to the design, conception, and completion of the study. MV, JS, and JT were responsible for providing the theoretical and methodological plan of the study. MV took lead in the data analysis and interpretation along with JS and AV. MV wrote the first drafts of the manuscript that was revised and modified by MH, JS, and JT. All authors contributed to the article and approved the submitted version.

## Conflict of Interest

The authors declare that the research was conducted in the absence of any commercial or financial relationships that could be construed as a potential conflict of interest.

## Footnotes

1 Pandemic-related behavior change does not refer to traditionally conceptualized behavior change, but rather self-assessments of the degree to which individuals have changed since the start of the pandemic.

2 The present study is part of a larger preregistered study examining age differences in thoughts, feelings, and reactions to the COVID19-pandemic. Currently, we are exploring pre-registered hypothesis 3 (the remaining pre-registered hypotheses are addressed in different manuscripts). The verbatim preregistered hypothesis was “Participants high in hostile ageism, compared to benevolent ageism, will self-report less fear of contracting COVID-19, greater endorsement of coronavirus humor, and less overall lifestyle changes as a result of this virus. This is a directional hypothesis.” In order to provide better clarity, interpretation, and focus to the current paper, we spilt the hypotheses so that benevolent and hostile ageism were presented on the two pandemic related variables, respectively. We re-worded the modified preregistered hypotheses presented here so that the expectations of hostile ageism focus on *lower* (rather than higher) hostile ageism. However, the pattern of expected findings was made to be identical to the pre-registered hypothesis by also switching the level of the dependent variables from higher fear to less fear and less behavioral change to more behavioral change. More information can be found on the preregistered project website (https://osf.io/ynbm3).

3 https://osf.io/ynbm3

4 One item from the pre-registered study assessing changes in airplane travel was removed from the behavior change scale due to lack of variability.

5 A dichotomized version of the variable assessing those who were in 100% agreement versus those with some doubt was explored but was not used as the pattern of effects using the dichotomized variable was comparable to the grouped variable. Dichotomizing variables risks losing interpretability and lowers statistical power.
