# Supplementary material for: Ageism and Behavior Change During a Health Pandemic: A Preregistered Study
Source: Front Psychol. 2020 Nov 19;11:587911. doi: 10.3389/fpsyg.2020.587911 (PMC7710520; doi:10.3389/fpsyg.2020.587911)
Supplement: Supplementary file 1 [file Table_1.DOCX]

**Table S1**

*Descriptive Statistics and Correlations for Ageism and Pandemic-Related Lifestyle Changes by Age Group*

| Variable | 1. | 2. | 3. | 4. | 5. | *M* | *SD* | *N* |
| --- | --- | --- | --- | --- | --- | --- | --- | --- |
| 1. Benevolent Ageism | – | .66^**^ | .08 | -.13^†^ | -.16^*^ | 2.10 | .98 | 171 |
| 2. Hostile Ageism | .69^**^ | – | .10 | -.07 | -.06 | 1.99 | .94 | 171 |
| 3.Pandemic Related Fear | .15^†^ | .05 | – | -.33^**^ | .29^**^ | 5.40 | 2.01 | 171 |
| 4. Behavior Change | -.21^**^ | -.26^**^ | .19^*^ | – | .45^**^ | 7.85 | .97 | 171 |
| 5. Social Distance Necessity | -.09 | -.11 | .33^**^ | .32^**^ | – | 91.52 | 17.15 | 171 |
| *M* | 2.87 | 2.80 | 5.73 | 7.26 | 82.43 |  |  |  |
| *SD* | 1.31 | 1.33 | 2.16 | 1.34 | 24.49 |  |  |  |
| *N* | 162 | 162 | 164 | 164 | 164 |  |  |  |

*Note.* Correlations among the older adults are above the diagonal line and the descriptive statistics for older adults are found in the columns right of the correlations. Correlations among the younger adults are below the diagonal line and their corresponding descriptive statistics are presented below the correlations. Transformed or rescaled data were used in the correlation matrix, and raw data were used for the means and standard deviations.

*^*^ p* < *.05,* ^**^*p* < .01, ^†^ *p* < .10

**Table S2**

*Tests of Moderation between Age Group and Ageism on Pandemic-Related Outcomes*

| Interaction | Age Group X  Benevolent Ageism | | | Age Group X  Hostile Ageism | | |
| --- | --- | --- | --- | --- | --- | --- |
|  | *b* | *se* | 95% CI | *b* | *se* | 95% CI |
| Pandemic-Related Fear | -.26 | .20 | [-.66, .14] | .05 | .21 | [-.36, .45] |
| Pandemic-Related Behavior Change | .03 | .10 | [-.17, .23] | .15 | .10 | [-.05, .35] |
| Social Distancing Necessity | -2.34 | 2.08 | [-6.44, .74] | .88 | 2.11 | [-3.28, 5.03] |

*Note*. Each moderation was conducted using Hayes’ (2013) PROCESS macro for SPSS. Model 1 was used to test basic moderation. *N* = 333 for each moderation analysis.

*^*^ p* < *.05,* ^**^*p* < .01, ^†^ *p* < .10
